# Transcriptional Comparison Reveals Differential Resistance Mechanisms between CMV-Resistant PBC688 and CMV-Susceptible G29

**DOI:** 10.3390/genes15060731

**Published:** 2024-06-02

**Authors:** Guangjun Guo, Baogui Pan, Chengsheng Gong, Shubin Wang, Jinbing Liu, Changzhou Gao, Weiping Diao

**Affiliations:** Jiangsu Key Laboratory for Horticultural Crop Genetic Improvement, Institute of Vegetable Crops, Jiangsu Academy of Agricultural Sciences, Nanjing 210014, China; ggj-198@163.com (G.G.); pantix@163.com (B.P.); 15738391652@163.com (C.G.); wangsbpep@163.com (S.W.); pepperljb@163.com (J.L.); gaochangzhou8@163.com (C.G.)

**Keywords:** pepper, *Cucumber mosaic virus*, transcriptome analysis, resistance mechanisms

## Abstract

The *Cucumber mosaic virus* (CMV) presents a significant threat to pepper cultivation worldwide, leading to substantial yield losses. We conducted a transcriptional comparative study between CMV-resistant (PBC688) and -susceptible (G29) pepper accessions to understand the mechanisms of CMV resistance. PBC688 effectively suppressed CMV proliferation and spread, while G29 exhibited higher viral accumulation. A transcriptome analysis revealed substantial differences in gene expressions between the two genotypes, particularly in pathways related to plant–pathogen interactions, MAP kinase, ribosomes, and photosynthesis. In G29, the resistance to CMV involved key genes associated with calcium-binding proteins, pathogenesis-related proteins, and disease resistance. However, in PBC688, the crucial genes contributing to CMV resistance were ribosomal and chlorophyll a–b binding proteins. Hormone signal transduction pathways, such as ethylene (ET) and abscisic acid (ABA), displayed distinct expression patterns, suggesting that CMV resistance in peppers is associated with ET and ABA. These findings deepen our understanding of CMV resistance in peppers, facilitating future research and variety improvement.

## 1. Introduction

The CMV is indeed a significant pathogen within the *Bromoviridae* family, particularly within the *Cucumovirus* genus. It holds prominence as one of the most prevalent viruses affecting plants globally [1]. More than 1200 plant species are known to be hosts for this virus, which is transmitted by over 80 species of aphids [2]. In pepper plants, CMV infection can lead to severe symptoms, including systemic mosaic patterns, dwarfism, and lesions on fruits. These manifestations often result in significant yield losses in affected regions annually [3,4]. The extensive host range and multitude of insect vectors pose considerable challenges to CMV control efforts. One promising approach to managing CMV is through genetic resistance in host plants. This strategy offers an environmentally friendly alternative to chemical interventions and avoids the complexities associated with transgenic techniques [5,6,7]. By breeding or selecting for resistance traits, researchers aim to develop pepper varieties that can withstand CMV infection more effectively, thereby mitigating its detrimental effects on yields.

While several accessions or varieties of *Capsicum annuum* [3,4,5,8,9] and *Capsicum frutescens* [9,10,11] have been found to be resistant to CMV, the genetic control of resistance is complex and largely unknown [6,7]. Mechanisms corresponding to three steps disrupting the virus cycle in plants have been shown to confer partial resistance to CMV in distinct *Capsicum* accessions: the restriction of virus installation in host cells [12,13], the restriction of virus proliferation in the whole plant [14], and restricting the long-distance movement of the virus [8,15]. These studies indicate that various resistant accessions possess different resistance mechanisms to CMV, and a single accession may also exhibit two or more resistance mechanisms. Understanding the resistance mechanism to CMV requires thorough molecular-level research due to the intricate resistance mechanisms of resistant accessions.

Unfortunately, a complete resistance capable of preventing CMV replication in peppers has not been found. Most of the identified sources exhibit partial resistance controlled by multiple genes, and several quantitative trait loci (QTLs) underlying resistance to CMV have been identified [5,8,11,16,17]. A single dominant resistance gene (*Cmr1*) has been reported to confer resistance to theCMV-P0 in the pepper variety ‘Bukang’ [4]. The C-terminus of the helicase domain encoded by CMV-P1 RNA1 has been demonstrated to determine susceptibility to systemic infection, controlling virus replication and cell-to-cell movement [18]. CMV resistance gene 2 (*cmr2*) from *C. annuum* landrace “Lam32” confers resistance to CMV-P1 in a recessive manner [6].

Although multiple QTLs and single genes for CMV resistance have been identified in peppers, the interaction mechanism of CMV with peppers, its influence on pepper gene expressions and biological processes, as well as CMV-triggered disease resistance remain unclear [6,11]. Therefore, the identification of various regulatory components involved in host–pathogen interactions can offer insights into the virus resistance mechanism and facilitate the development of appropriate molecular markers for breeding purposes [19]. Because the complexity of these responses, which involve numerous physiological processes, transcriptomic studies prove valuable in comprehensively understanding the plant’s reactions to viruses [20]. RNA sequencing (RNA-seq) is the most powerful tool for transcriptome characterization in investigations of disease resistance in plants. RNA-seq allows gene discovery and expression profiling, aiding in the identification of key components of resistance pathways [21]. Several studies have investigated the genome-wide expression profiles of plants after CMV infection, as well as the molecular mechanisms in the pathogens associated with host infections, such as Arabidopsis [22], chrysanthemum [23], tobacco [21], and hot peppers [7].

This study delved into the response of CMV-resistant (PBC688) and – CMV-susceptible (G29) pepper accessions to CMV infection. Leaves were harvested from both accessions during two treatments, CK (blank control for phosphate buffer inoculation) and 14 days post-inoculation with CMV, for subsequent analysis. A quantitative reverse transcription polymerase chain reaction (qRT-PCR) was employed to determine the CMV copy numbers in the leaves, while RNA-seq analysis was utilized to examine the gene expression profiles. A comparative assessment of the RNA-seq datasets between the resistant and susceptible genotypes unveiled noteworthy disparities in the expression of genes associated with various biological processes, including plant–pathogen interactions, mitogen-activated protein kinase signaling, ribosome function, and photosynthesis–antenna proteins. These findings contribute novel insights into the genetic mechanisms governing resistance against CMV in pepper plants. 

## 2. Materials and Methods

### 2.1. Plant Materials 

This study utilized two pepper accessions: *C. frutescens* cv. PBC688, known for its high resistance to CMV, and the susceptible accession *C. annuum* cv. G29. These plants were cultivated in the artificial climate chamber of the Jiangsu Academy of Agricultural Sciences, with a temperature maintained at 25 °C for 16 h of lighting and 20 °C for 8 h of darkness. 

### 2.2. Virus Strains and Inoculation Method

The CMV isolate utilized in this study was CMV_FNY_, which was supplied by Dr. Ji from the Institute of Plant Protection, Jiangsu Academy of Agricultural Sciences. Seedlings were inoculated with CMV_FNY_ when three leaves were fully expanded, and the appearance of a new leaf was noted. The CMV_FNY_ inoculum was prepared from infected leaves of *Nicotiana benthamiana*. One gram of infected leaves was ground in 5 mL of a 0.02 M phosphate buffer with a pH of 7.0. Pepper plants were dusted with 600-mesh carborundum and then inoculated by rubbing the leaves with the prepared inoculum. Subsequently, pepper seedlings were kept in a greenhouse which was maintained at temperatures ranging from 25 to 30 °C. Symptoms of CMV infection were initially observed one-week post-inoculation, with continuous monitoring of symptom development until two weeks post-inoculation.

### 2.3. Absolute Quantification of CMVs Copy Number

#### 2.3.1. Sampling 

The sampling procedure involves collecting newly expanded leaves from one plant for each genotype (PBC688 and G29). This sampling is conducted during two treatments: a blank control for phosphate buffer inoculation (CK) and 14 days after CMV inoculation. The purpose is to determine the CMV virus copy number to detect virus proliferation. Additionally, five consecutive leaves starting from the inoculated leaf upwards are sampled to measure the CMV virus content for detecting the long-distance movement of the virus. The sampling was conducted in triplicate to ensure reliability and reproducibility of the results.

#### 2.3.2. RNA Extraction and cDNA Preparation

The primers were designed based on the CMV CP gene sequence registered in GenBank (Registry Number: AY965892.1). The forward primer, CMV-cp-F, has the sequence TTCCGCTTCCTACCGTTCTAT, while the reverse primer, CMV-cp-R, has the sequence CGAACCAATCTGTATCGTCAAA. These primers were synthesized by Sangon Biotech (Shanghai, China).

Total RNAs from all samples were extracted using the TaKaRa MiniBEST Plant RNA Extraction Kit (TaKaRa, Shiga, Japan). RNA integrity and purity were assessed via agarose gel electrophoresis to monitor degradation and contamination.

Reverse transcription was carried out using the Hifair^®^ III 1st Strand cDNA Synthesis SuperMix for qPCR (gDNA digester plus) from YEASEN (Shanghai, China). This process involved converting RNA into complementary DNA (cDNA), which served as the template for a subsequent quantitative PCR (qPCR) analysis.

#### 2.3.3. Plasmid Standard Preparation

The plasmid standard was prepared following the method outlined below. Initially, the target fragment was amplified by PCR using cDNA as templates. The reaction mixture consisted of 12.5 µL of 2× PCR Master Mix, 0.5 µL of CMV-cp-F (10 µmol), 0.5 µL of CMV-cp-R (10 µmol), and 1 µL of the cDNA template and sterile water added to a final volume of 25 µL. The PCR amplification was performed under the following conditions: initial denaturation at 94 °C for 3 min, followed by 35 cycles of denaturation at 94 °C for 10 s, annealing at 60 °C for 10 s, extension at 72 °C for 30 s, and a final extension at 72 °C for 10 min. The amplified products were visualized by agarose gel electrophoresis.

Next, the target fragments were extracted using the AxyPrepTM DNA Gel Extraction Kit (Axygen, Tewksbury, MA, USA), ligated into the pMD19-T vector, and transformed into JM109 cells. White colonies were selected using blue–white selection, and the plasmids were isolated using the FastPure Plasmid Mini Kit (Vazyme, Nanjing, China). The plasmid with correct sequencing was used as the positive control. The concentration of the plasmid was determined using NanoDrop 2000C (Thermol Fisher, Waltham, MA, USA), and then the CMV copy number was calculated using the following formula:

$$CMV\;copy\;number=\frac{6.02\times 10^{23}\times plasmid\;concentration\;(\frac{ng}{\mu L})\times 10^{-9}}{the\;base\;number\;of\;plasmid\;(bp)\times 660}$$

The calculated copy number of the plasmid was 3.4686 × 10^10^ μL^−1^. The plasmid DNA was then diluted in a 10-fold gradient to serve as the standard for absolute quantification.

#### 2.3.4. Calculation of the CMV Copy Number in Samples

The number of copies of the CMV in the unknown sample can be determined by substituting the Ct value of the unknown sample into the resulting standard curve. The Ct value represents the number of cycles that the fluorescence signal in each reaction tube undergoes before reaching the set threshold value. By comparing the Ct value of the unknown sample with the Ct values of the standards on the standard curve, the corresponding number of copies of CMV can be interpolated or extrapolated. This allows for the quantification of CMV in the unknown sample based on its Ct value in the PCR reaction.

### 2.4. cDNA Library Construction and Sequencing

The experiment involved sampling fully expanded leaves from the top of plants, with three plants sampled for each genotype at two treatment. CK and 14 days after CMV inoculation. RNA extraction and cDNA library construction were performed on these samples, with three independent duplicates conducted. In total, there were 12 qualified samples (2 genotypes × 2 time points × 3 duplicates), all of which were sent to Novogene Bioinformatics Technology Co., Ltd., in Beijing, China, for RNA-Seq analysis. 

Before sequencing, the RNA concentration was precisely measured using a Qubit^®^ 2.0 Fluorometer (Life Technologies, Carlsbad, CA, USA), and the RNA integrity was assessed using an Agilent 2100 Bioanalyzer system (Agilent Technologies, Santa Clara, CA, USA). All samples had a minimum RNA integrity number (RIN) of 7.2, indicating their suitability for sequencing.

The mRNA from each sample was enriched using magnetic beads with Oligo(dT), which specifically binds to the poly(A) tail of mRNA through A–T complementary pairing. Subsequently, the mRNA was fragmented using a fragmentation buffer. The fragmented mRNA served as a template for cDNA synthesis, with random hexamer primers utilized to synthesize the first cDNA strand. The second cDNA strand was synthesized by adding a buffer, dNTPs, and DNA polymerase I.

The synthesized double-stranded cDNA was purified using AMPure XP beads (Beckman Coulter, Brea, CA, USA), followed by end repair and A-tailing. Sequencing adapters were then ligated to the A-tailed cDNA fragments. Fragment size selection was performed using AMPure XP beads, and PCR enrichment was conducted to obtain the final cDNA libraries.

The concentration of libraries was initially quantified using the Qubit 2.0 system and then diluted to 1 μg/μL for assessing the insert size using the Agilent 2100 Bioanalyzer. Once the insert size met expectations, accurate library quantification was performed using Q-PCR, with a concentration higher than 2 nM considered adequate.

The 12 qualified cDNA libraries were pooled together and sequenced on an Illumina HiSeq 2500 platform at Novogene Bioinformatics Technology Co., Ltd. (Beijing, China) using 150 bp paired-end sequencing. The raw paired-end reads were deposited into the Sequence Read Archive of the NCBI with the BioProject accession number PRJNA1110809.

### 2.5. RNA-Seq Data Analysis

#### 2.5.1. Quality Control

Raw data (raw reads) of fastq format were first processed through fastp (v0.19.4) In this step, clean data (clean reads) were obtained by removing reads containing an adapter, reads containing ploy-N, and low quality reads from the raw data. At the same time, the Q20, Q30, and GC content of the clean data were calculated. All the downstream analyses were based on clean data with high quality. 

#### 2.5.2. Reads Mapping to the Reference Genome

Reference genome and gene model annotation files were directly downloaded from the genome website [https://ftp.ensemblgenomes.ebi.ac.uk/pub/plants/release-56/fasta/capsicum_annuum/dna/Capsicum_annuum.ASM51225v2.dna.toplevel.fa.gz (accessed on 17 February 2023 )] and [https://ftp.ensemblgenomes.ebi.ac.uk/pub/plants/release-56/gtf/capsicum_annuum/Capsicum_annuum.ASM51225v2.56.gtf.gz (accessed on 14 January 2023)]. An index of the reference genome was built using Hisat2 v2.0.5, and paired-end clean reads were aligned to the reference genome using Hisat2 v2.0.5 [24]. We selected Hisat2 as the mapping tool, since Hisat2 can generate a database of splice junctions based on the gene model annotation file and thus a better mapping result than other non-splice mapping tools.

#### 2.5.3. Novel Transcript Prediction

The mapped reads of each sample were assembled by StringTie (v1.3.3b) [24,25] in a reference-based approach. StringTie uses a novel network flow algorithm as well as an optional de novo assembly step to assemble and quantitate full-length transcripts representing multiple splice variants for each gene locus.

#### 2.5.4. Quantification of Gene Expression Levels

To quantify gene expression levels, feature Counts v1.5.0-p3 was used to count the reads numbers mapped to each gene [26]. And then, the FPKM (Fragments Per Kilobase of transcript sequence per Million base pairs sequenced) of each gene was calculated based on the length of the gene and reads count mapped to this gene. Based on the FPKM expression profiles of all genes across the samples, a PCA analysis was conducted using the Cloud Platform for After-sales Service of Novogene Bioinformatics Technology Co., Ltd., Beijing, China. (https://magic.novogene.com/customer/main#/homeNew (accessed on 19 August 2021)). 

#### 2.5.5. Differential Expression Analysis

A differential expression analysis was performed using the DESeq2 R package (1.20.0) [27]. The resulting *p* values were adjusted using Benjamini and Hochberg’s approach for controlling the false discovery rate. Genes with an adjusted *p* value of ≤ 0.05, as found by DESeq2, were assigned as differentially expressed. Prior to the differential gene expression analysis, for each sequenced library, the read counts were adjusted by the edgeR program package through one scaling normalized factor. A differential expression analysis of two conditions was performed using the edgeR R package (3.22.5) [28]. The *p* values were adjusted using the Benjamini and Hochberg method. A corrected *p* value of 0.05 and an absolute fold change of 2 were set as the threshold for a significantly differential expression. 

#### 2.5.6. GO and KEGG Enrichment Analysis of Differentially Expressed Genes

A gene ontology (GO) enrichment analysis of differentially expressed genes was implemented by the cluster Profiler R package, in which the gene length bias was corrected [29]. GO terms with a corrected p value of less than 0.05 were considered significantly enriched by differentially expressed genes. KEGG is a database resource for understanding high-level functions and utilities of the biological system, such as the cell, the organism, and the ecosystem, from molecular-level information, especially large-scale molecular datasets generated by genome sequencing and other high through-put experimental technologies (http://www.genome.jp/kegg/ (accessed on 1 April 2023)) [30]. We used the cluster Profiler R package to test the statistical enrichment of differentially expressed genes in KEGG pathways [31]. 

#### 2.5.7. Heatmap Analysis of Differentially Expressed Genes

For the visualization of target gene FPKM values, we employed the pheatmap package to generate heatmaps. Specific scripts for this process can be found at the following link: https://zhuanlan.zhihu.com/p/617854543?utm_id=0 (accessed on 26 October 2023). First, we normalize the data of all genes using a log^2^(X + 1) transformation. Then, we standardize the normalized data using the Z-score normalization method, also known as zero-mean normalization. After this process, the distribution of the data conforms to a standard normal distribution, with a mean of 0 and a standard deviation of 1. The formula for standardization is as follows: z = (x − μ)/σ. Here, μ represents the mean of all sample data, andσrepresents the standard deviation of all sample data. In this formula, the Z value represents the distance between the original score and the population mean, calculated in units of standard deviation. Z is negative when the original score is below the mean and positive otherwise. Finally, based on the relative distances between genes, we divided them into different subclusters to achieve a clustering analysis. 

This comprehensive analysis pipeline aimed to identify and characterize genes and pathways associated with the plant’s response to CMV infection and provide insights into the molecular mechanisms underlying genetic resistance to the virus.

## 3. Results

### 3.1. Absolute Quantification of CMV Copies in CMV-Susceptible G29 and CMV-Resistant PBC688

The standard curve for CMV was established via RT-PCR using a gradient-diluted CMV plasmid as a template. The copy number of the standard template ranged from 3.4686 × 10^3^ to 3.4686 × 10^8^ μL^−1^, showing a strong linear relationship with the Ct value. The primer amplification efficiency was 110.08%. (A Ct value of >30 was considered negative in this experiment.) (Supplementary Figure S1 and Table S1.)

**Figure 1:**
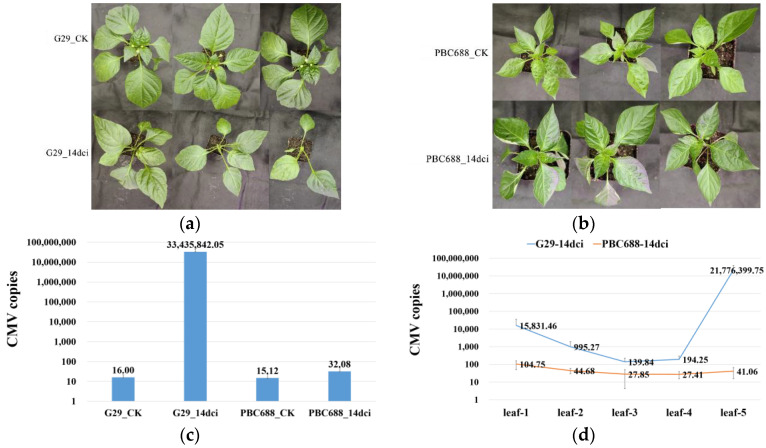
Phenotypic characteristics and the copy number of CMV in leaves of CMV-resistant PBC688 and CMV-susceptible G29. (**a**) Phenotypic symptoms after 14 days of phosphate buffer inoculation (CK) and CMV infection (14dci) in CMV-susceptible G29; (**b**) phenotypic symptoms before and after 14 days of phosphate buffer inoculation (CK) and CMV infection (14dci) in CMV-resistant PBC688; (**c**) CMV copy number in apical leaves of G29 and PBC688 CK and 14dci; (**d**) CMV copy number in the five consecutive leaves starting from the inoculated leaf (leaf-1) of G29_14dci and PBC688_14dci.

Fourteen days post-CMV inoculation, G29 displayed symptoms including leaf yellowing, severe systemic phloem, and growth point necrosis compared to the G29-CK (Figure 1a). Conversely, PBC688 showed no discernible differences compared to the PBC688-CK and remained asymptomatic (Figure 1b).

An absolute quantitative analysis of CMV copies revealed that the CMV copy numbers in the apical leaves of the G29 and PBC688 control plants were 16.00 and 15.12 copies/µL, respectively, with no significant difference between them. However, the CMV copy number in the apical leaves of the CMV-inoculated G29 was 3.34 × 10^8^ ± 1.21 × 10^8^ copies/µL, whereas in the CMV-inoculated PBC688, it was only 32.08 ± 16.15 copies/µL.

The disparity between the CMV-resistant PBC688 and the CMV-susceptible G29 was highly significant (Figure 1c and Table S2). 

A quantification of CMV copies in the five consecutive leaves starting from the inoculated leaf demonstrated that the CMV copies in the whole plant of G29 was significantly higher than that of PBC688. The highest CMV copies of 2.18 × 10^8^ ± 1.74 × 10^8^ copies/µL was observed in the apical leaf of G29-14dci, whereas in the inoculated leaf, it was 1.58 × 10^5^ ± 1.97 × 10^5^ copies/µL. In contrast, the second, third, and fourth leaves exhibited 995.27 ± 956.08 copies/µL, 139.84 ± 83.68copies/µL, and 194.25 ± 99.11 copies/µL, respectively. The CMV copies of the inoculated leaf in PBC688-14dci was highest at 104.75 ± 54.23 copies/µL, while the CMV copies of the other four leaves did not exceed 50 copies/µL (Figure 1d and Table S3). 

These findings suggest that PBC688 significantly inhibited the proliferation of CMV in the cells and partially inhibited the long-distance movement of CMV. However, in the CMV-susceptible G29, there was significant CMV proliferation, along with the capacity for long-distance transport, ultimately resulting in the highest accumulation of viral content in the newly emerging apical leaves.

### 3.2. Transcription Profile of CMV-Susceptible G29 and CMV-Resistant PBC688

The transcriptomes of two pepper accessions during targeted treatments of CK and 14dci were sequenced, yielding 77.1 G clean reads and 6.43 G high-quality bases per sample after sequence filtration. On average, 86.85% of the clean reads from each sample uniquely aligned to the pepper reference genome, with the percentage of Q30 bases at 91.96% or above (Table S4). The sequencing data exhibited sufficient quantity and high quality, making them suitable for further analysis.

**Figure 2:**
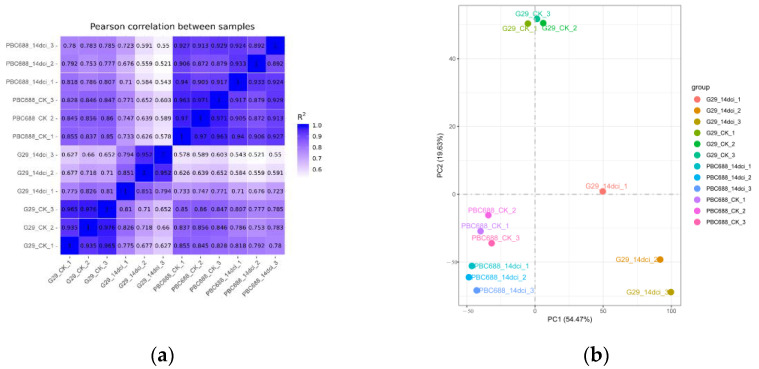
Pearson analysis and principal component analysis (PCA) among CMV-susceptible G29 and CMV-resistant PBC688 uninoculated with CMV (CK) and 14 days after CMV inoculation (14dci). (**a**) Pearson analysis among CMV-susceptible G29 and CMV-resistant PBC688 uninoculated with CMV (CK) and 14 days after CMV inoculation (14dci); (**b**) principal component analysis (PCA) among CMV-susceptible G29 and CMV-resistant PBC688 uninoculated with CMV (CK) and 14 days after CMV inoculation (14dci).

A total of 44,432 genes were identified through sequence alignment, including 8587 novel genes. FPKM values were utilized as the normalized expression values of genes, revealing that the expression levels of most genes fell within the range of 0.1 to 10. A Pearson correlation analysis demonstrated that the correlation coefficients for the three replicates of G29_CK, PBC688_CK, G29_14dci, and PBC688_14dci were greater than 0.91, 0.95, 0.794, and 0.892, respectively (Figure 2a). Alternatively, a principal component analysis of all genes revealed that the first two principal components accounted for 57.47% and 19.63% of the variance, respectively. Notably, significant separation among different treatments was observed. Specifically, the three biological replicates of G29_CK, PBC688_CK, and PBC688_14dci clustered together, while the three biological replicates of G29_14dci were more dispersed (Figure 2b).

These findings suggest significant differences in gene expression levels in the leaf tissues of PBC688_CK and G29_CK. Furthermore, the gene expression levels of PBC688_14dci were highly similar to those of PBC688_CK, whereas G29_14dci exhibited notable differences in gene expression levels following CMV inoculation.

### 3.3. Transcriptional Response of CMV-Susceptible G29 and CMV-Resistant PBC688

**Figure 3:**
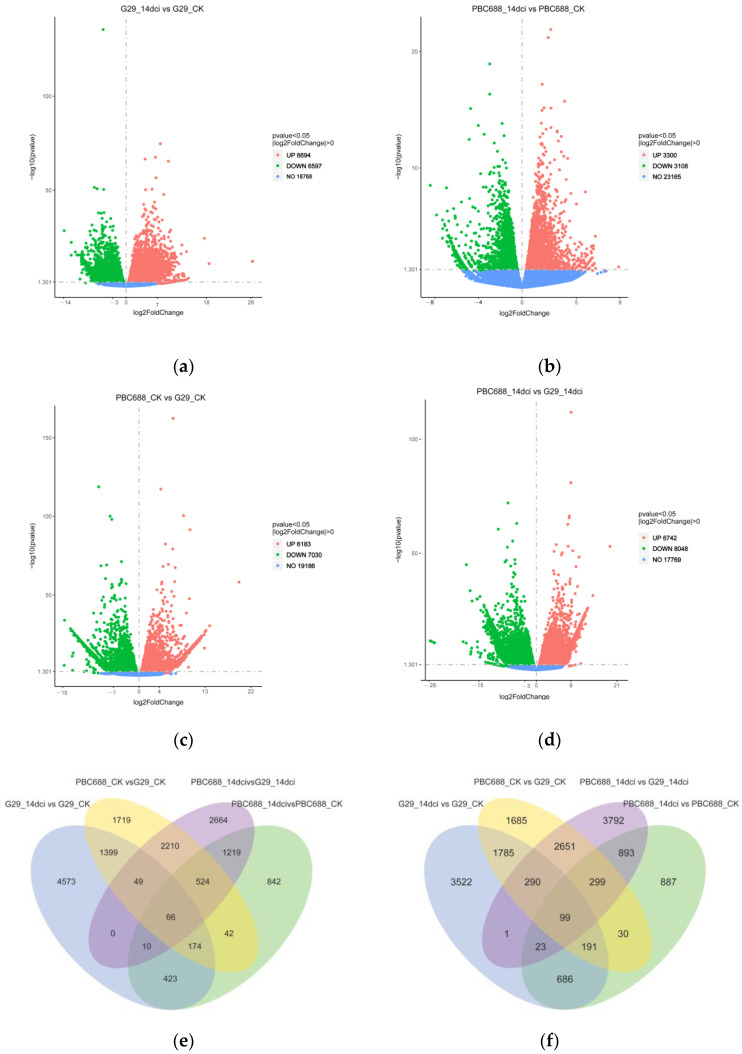
Differentially expressed genes between different treatments and Venn analysis of differentially expressed genes. (**a**) The differentially expressed genes between G29_14dci and G29_CK; (**b**) the differentially expressed genes between PBC688_14dci and PBC688_CK; (**c**) the differentially expressed genes between PBC688_CK and G29_CK; (**d**) the differentially expressed genes between PBC688_14dci and G29_14dci; (**e**) Venn analysis of upregulated differentially expressed genes; (**f**) Venn analysis of down-regulated differentially expressed genes.

To identify differentially expressed genes (DEGs), pairwise comparisons were conducted between pre-inoculation and post-inoculation with CMV within the CMV-susceptible G29 and the CMV-resistant PBC688. A total of 13,291 DEGs were identified between G29_14dci and G29_CK, with 6694 upregulated and 6597 downregulated genes. Similarly, 6408 DEGs were identified between PBC688_14dci and PBC688_CK, consisting of 3300 upregulated and 3108 downregulated genes (Figure 3a,b; Tables S5 and S6). These findings reveal that the number of DEGs in G29_14dci vs G29_CK was more than twice that in PBC688_14dci vs PBC688_CK, suggesting a more pronounced impact of CMV infestation on the immune system of the CMV-susceptible G29. Furthermore, there were 13,213 DEGs between PBC688_CK and G29_CK, among which 6183 were upregulated and 7030 were downregulated. Similarly, there were 14,790 DEGs between PBC688_14dci and G29_14dci, with 6742 upregulated and 8048 downregulated genes (Figure 3c,d; Tables S7 and S8). These results indicate that the disparity in gene expression levels between PBC688_CK and G29_CK was more substantial than the influence of CMV infestation on the gene expression levels of PBC688 and G29.

To gain a deeper understanding of the response of the CMV-resistant PBC688 and the CMV-susceptible G29 to CMV infection, a Venn analysis of the DEGs was performed. After CMV inoculation, 6694 and 3300 genes were upregulated in PBC688_14dci and G29_14dci, respectively, with 673 genes co-upregulated in both. Relative to G29_CK and G29_14dci, 6183 and 6742 genes were upregulated in PBC688_CK and PBC688_14dci, respectively, with 2849 genes co-upregulated in both. Additionally, 125 genes were co-upregulated between PBC688_14dci vs G29_14dci and G29_14dci vs G29_CK, 1819 genes were co-upregulated between PBC688_14dci vs G29_14dci and PBC688-14dci vs PBC688-CK, and 76 genes were co-upregulated among PBC688_14dci vs G29_14dci, G29_14dci vs G29_CK, and PBC688-14dci vs PBC688-CK (Figure 3e).

Similarly, after CMV inoculation, 3109 and 6597 genes were downregulated in PBC688_14dci and G29_14dci, respectively, with 1000 genes co-downregulated in both. Compared to G29_CK and G29_14dci, 7030 and 8049 genes were downregulated in PBC688_CK and PBC688_14dci, respectively, with 3339 genes co-downregulated in both. In total, 414 genes were co-downregulated between PBC688_14dci vs G29_14dci and G29_14dci vs G29_CK, 1315 genes were co-downregulated between PBC688_14dci vs G29_14dci and PBC688-14dci vs PBC688-CK, and 123 genes were co-downregulated among PBC688_14dci vs G29_14dci, G29_14dci vs G29_CK, and PBC688-14dci vs PBC688-CK (Figure 3f).

### 3.4. GO Annotation and Enrichment Analysis of DEGs

To elucidate the functional roles of genes associated with CMV resistance in the CMV-susceptible G29 and the CMV-resistant PBC688, we conducted a gene ontology (GO) analysis using the identified DEGs across three main categories: biological process, cellular component, and molecular function (adjusted *p* value of <0.05).

**Figure 4:**
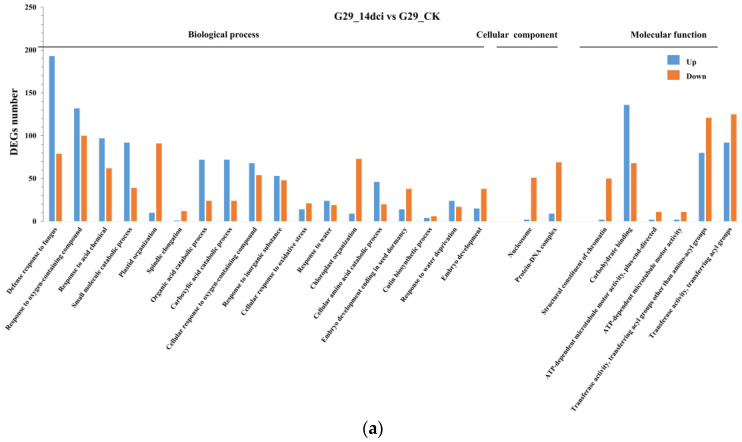

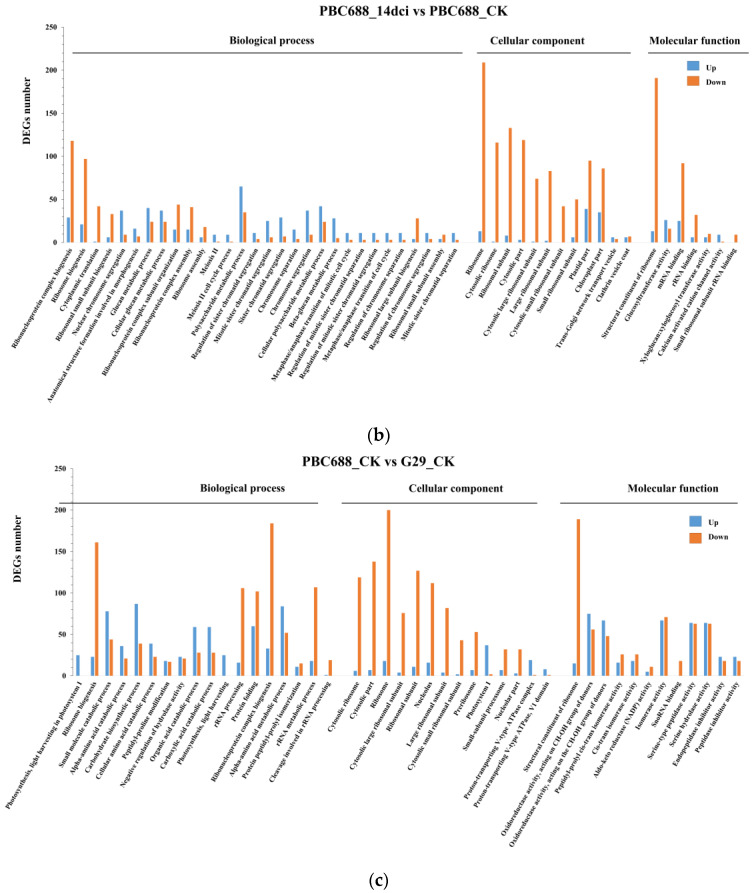

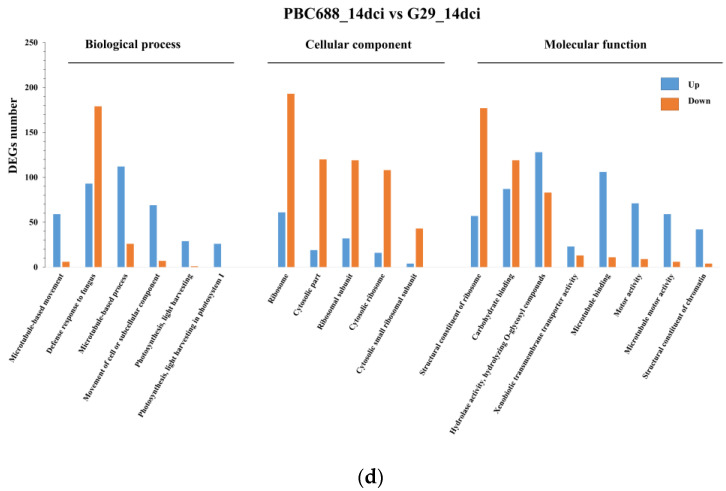
Gene ontology (GO) terms associated with differentially expressed genes between different treatments. (**a**) GO annotation and enrichment analysis of DEGs between G29_14dci and G29_CK; (**b**) GO annotation and enrichment analysis of DEGs between PBC688_14dci and PBC688_CK; (**c**) GO annotation and enrichment analysis of DEGs between PBC688_CK and G29_CK; (**d**) GO annotation and enrichment analysis of DEGs between PBC688_14dci and G29_14dci.

The DEGs between G29_14dci and G29_CK, PBC688_14dci and PBC688_CK, PBC688_CK and G29_CK, and PBC688_14dci and G29_14dci were linked to 26, 49, 44, and 19 biological processes, cellular components, and molecular functions, respectively (Figure 4).

In G29_14dci vs G29_CK, DEGs were predominantly associated with biological processes related to stress responses to biotic and abiotic factors such as fungi, acids, and oxygenated compounds (Figure 4a). DEGs between PBC688_14dci and PBC688_CK were enriched in processes like ribosome biogenesis and function, meiosis II cell cycle progression, and chromosome separation (Figure 4b). The DEGs between PBC688_CK and G29_CK were linked to processes such as photosynthesis, ribosome biogenesis, small molecule metabolism, and amino acid biosynthesis (Figure 4c). DEGs between PBC688_14dci and G29_14dci were associated with biological processes including microtubule-based movement, defense response to fungi, and light harvesting in photosynthesis. Cellular components such as ribosomes and cell membrane constituents, as well as molecular functions like structural components of ribosomes, carbohydrate binding, xenobiotic transmembrane transport, and microtubule movement, were also highlighted (Figure 4d).

Further analysis revealed that DEGs involved in ribosome function (GO: 0005840) and fungal defense (GO:0050832) were predominantly downregulated in the CMV-resistant PBC688_14dci compared to the CMV-susceptible G29_14dci. Conversely, DEGs associated with microtubule function (GO:0007017, GO:0008017, and GO:0003777) and photosynthesis (GO:0009765 and GO:0009768) exhibited a mostly upregulated expression in PBC688_14dci, particularly with 56 out of 55 DEGs related to photosynthesis being upregulated and one being downregulated (Table S9). 

These results suggest that CMV-resistant genes in PBC688 were primarily associated with ribosome function, microtubule movement, and photosynthesis, while showing less correlation with biotic stress responses.

### 3.5. KEGG Functional Annotations of DEGs

**Figure 5:**
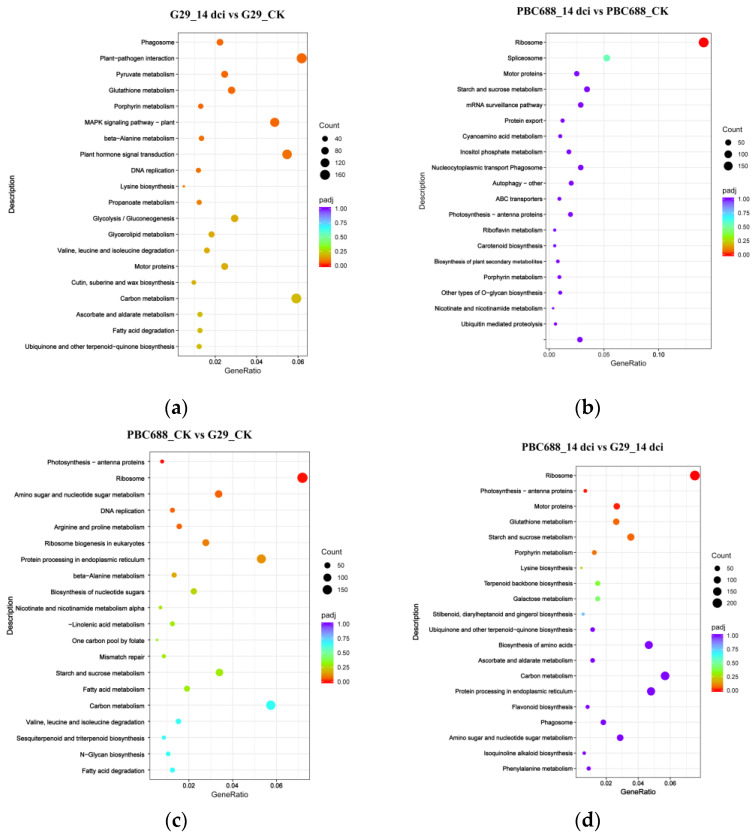
The top 20 Kyoto Encyclopedia of Genes and Genomes (KEGG) pathways with the highest representation of differentially expressed genes (DEGs) between different treatments. (**a**) The top 20 KEGG pathways of DEGs between G29_14dci and G29_CK; (**b**) the top 20 KEGG pathways of DEGs between PBC688_14dci and PBC688_CK; (**c**) the top 20 KEGG pathways of DEGs between PBC688_CK and G29_CK; (**d**) the top 20 KEGG pathways of DEGs between PBC688_14dci and G29_14dci.

In addition to a GO analysis, the DEGs from the four comparisons were subjected to KEGG enrichment pathway mapping, with encoded enzymes assigned to 123 KEGG pathways in G29_14dci vs G29_CK (Table S10), 121 KEGG pathways in PBC688_14dci vs PBC688_CK (Table S11), 123 KEGG pathways in PBC688_CK vs G29_CK (Table S12), and 123 KEGG pathways in PBC688_14dci vs G29_14dci (Table S13). The top 20 KEGG pathways with the highest representation of DEGs are presented in Figure 5.

In G29_14dci vs G29_CK, no significantly enriched pathway was observed at a padj ≤ 0.05 threshold (Figure 5a). Conversely, in PBC688_14dci vs PBC688_CK, the ribosome pathway was significantly enriched (Figure 5b). For PBC688_CK vs G29_CK, the significantly enriched pathways included photosynthesis and ribosomes (Figure 5c). Notably, in PBC688_14dci vs G29_14dci, the majority of DEGs were implicated in pathways such as ribosomes and photosynthesis (Figure 5d). These results underscore the differential pathway enrichment of DEGs in the CMV-susceptible G29 and the CMV-resistant PBC688 following CMV infection, indicating distinct molecular responses between the two genotypes.

### 3.6. Annotation of DEGs in Response to CMV Infection of G29

The KEGG analysis revealed the identification of 165 DEGs associated with plant–pathogen interactions in G29_14dci vs G29_CK. Among these, 106 DEGs exhibited upregulation, while 59 DEGs showed downregulation in G29_14dci. Similarly, 131 DEGs were enriched in the MAPK signaling pathway, with 101 DEGs upregulated and 30 DEGs downregulated in G29_14dci (Tables S9–S11). 

**Figure 6:**
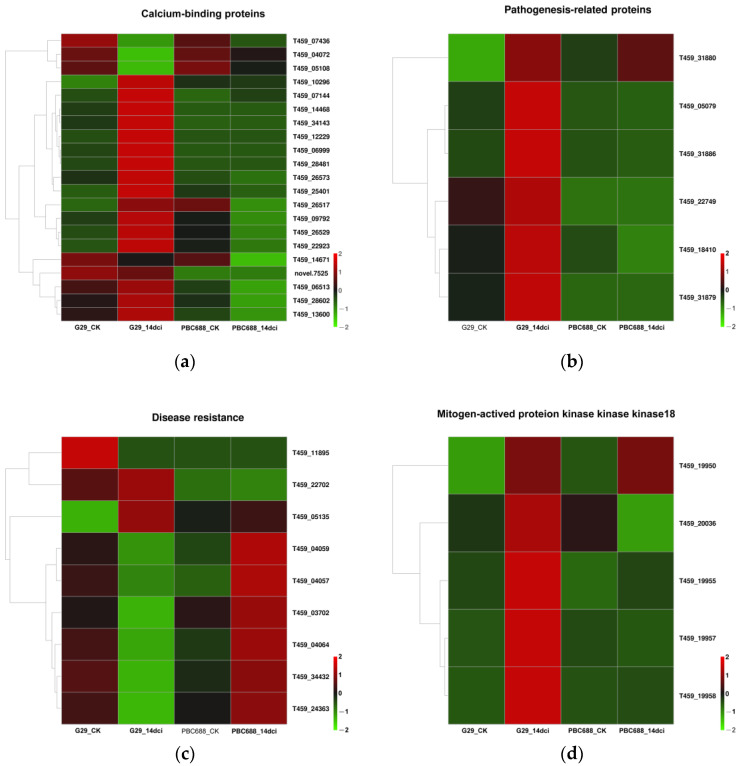
Heatmaps of the differentially expressed genes (DEGs) associated with plant–pathogen interactions and the MAPK signaling pathway. (**a**) Heatmaps of the DEGs associated with calcium-binding proteins; (**b**) heatmaps of the DEGs associated with pathogenesis-related proteins; (**c**) heatmaps of the DEGs associated with disease resistance; (**d**) heatmaps of the DEGs associated with mitogen-activated protein kinase kinase kinase 18.

In the DEGs associated with plant–pathogen interactions and the MAPK signaling pathway, several genes encoding disease-resistant proteins exhibited differential expressions. Specifically, there were 21, 6, 9, and 5 DEGs associated with calcium-binding proteins (Figure 6a), pathogenesis-related proteins (PRs) (Figure 6b), disease resistance (Figure 6c), and mitogen-activated protein kinase kinase kinase 18 (MAPKKK18) (Figure 6d), respectively. Following CMV infection, the majority of DEGs were upregulated in G29 but downregulated in PBC688. However, the expression levels of the 9 DEGs related to disease resistance were relatively low. Post-CMV infection, only 2 DEGs were upregulated in G29, whereas 6 DEGs were upregulated in PBC688 (Figure 6c).

**Figure 7:**
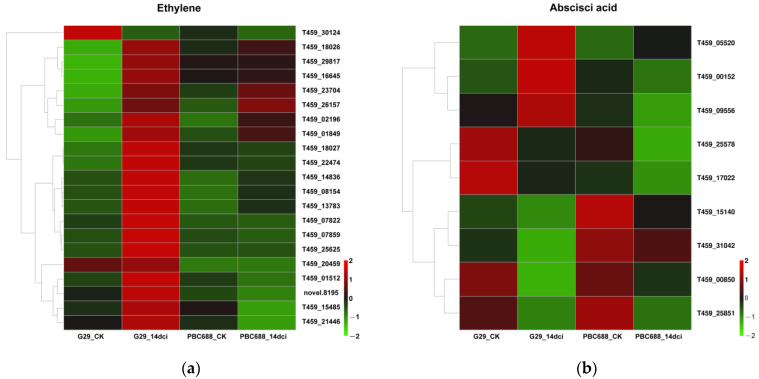
Heatmaps of the differentially expressed genes (DEGs) associated with signal transduction pathways. (**a**) Heatmaps of the DEGs associated with ethylene (ETH) pathway; (**b**) heatmaps of the DEGs associated with abscisic acid (ABA) pathway.

In the DEGs associated with plant–pathogen interactions and the MAPK signaling pathway, approximately 30 DEGs involved in hormone signal transduction were identified, including 21 DEGs involved in the ethylene pathway and 9 DEGs involved in the ABA pathway (Figure 7). Among these, 20 DEGs encoding ethylene were significantly upregulated following CMV infection in G29, while only 2 of the 21 DEGs were significantly upregulated following CMV infection in PBC688 (Figure 7a). Additionally, three DEGs (T459_05520, T459_00152, and T459_09556) involved in the ABA pathway were upregulated following CMV infection in G29 (Figure 7b). This differential expression pattern suggested a potential role for the ethylene pathways in the response to CMV infection, particularly in the G29 genotype.

### 3.7. Annotation of DEGs in Response to CMV Infection of PBC688

**Figure 8:**
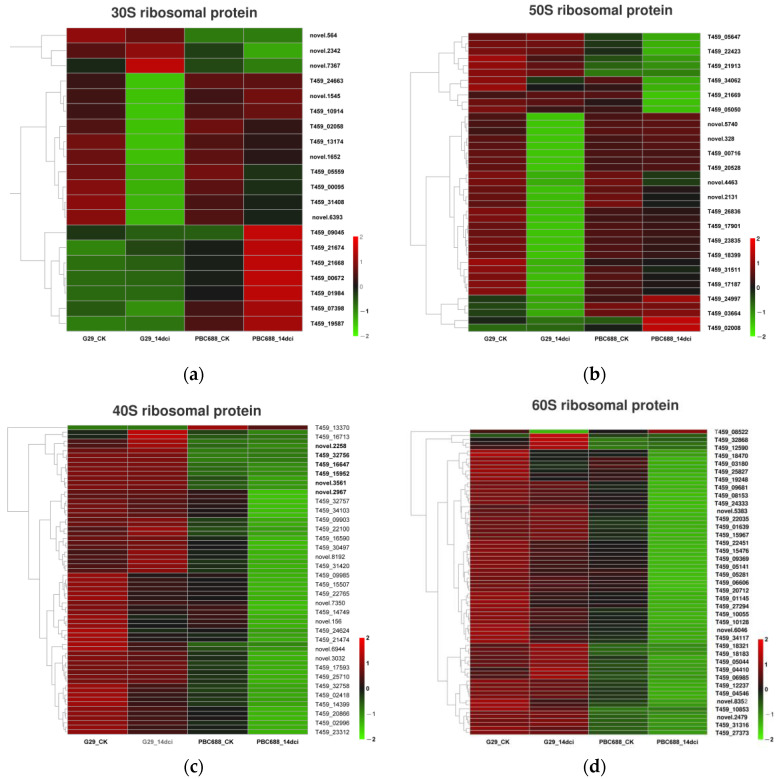
Heatmaps of the differentially expressed genes (DEGs) associated with ribosome pathway. (**a**) Heatmaps of the DEGs associated with 30S ribosomal proteins; (**b**) heatmaps of the DEGs associated with 50S ribosomal proteins; (**c**) heatmaps of the DEGs associated with 40S ribosomal proteins; (**d**) heatmaps of the DEGs associated with 60S ribosomal proteins.

In the KEGG analysis, 215 DEGs were found to be enriched in the ribosome pathway in PBC688_14dci compared to G29_14dci, with 52 upregulated and 163 downregulated DEGs in PBC688_14dci. Among these DEGs, 20, 67, 41, and 77 were associated with 30S, 40S, 50S, and 60S ribosomal proteins, respectively. Notably, while most DEGs linked to 30S and 50S ribosomal proteins in the CMV-resistant PBC688 did not show significant differences compared to the CMV-susceptible G29, a substantial proportion of DEGs associated with 40S and 60S ribosomal proteins exhibited lower expression levels in PBC688. Following CMV infection, the majority of DEGs associated with 30S and 50S ribosomal proteins were downregulated in G29, whereas some were upregulated in PBC688 (Figure 8a,b). Conversely, most DEGs associated with 40S and 60S ribosomal proteins were significantly downregulated in PBC688_14dci, while a portion of them were upregulated in G29_14dci (Figure 8c,d). These findings indicate a potential role of ribosomal protein regulation in the response to CMV infection, with complex dynamics observed following viral exposure. 

**Figure 9:**
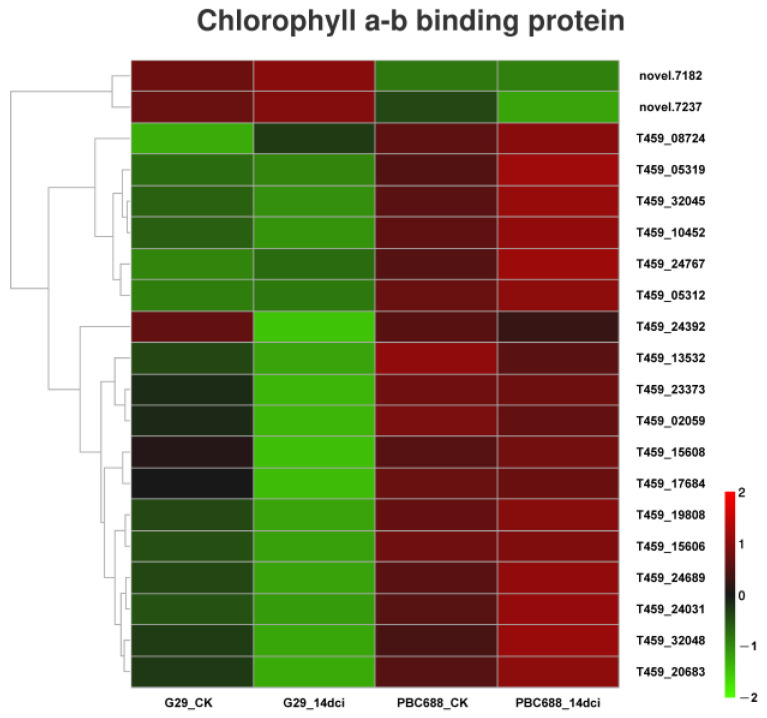
Heatmaps of the differentially expressed genes (DEGs) associated with chlorophyll a–b binding proteins.

In the photosynthesis–antenna proteins pathway, all 20 differentially expressed genes (DEGs) encode chlorophyll a–b binding proteins. Most of these DEGs in the CMV-resistant PBC688 exhibited significant differences compared to the CMV-susceptible G29. After CMV inoculation, 14 DEGs showed significantly increased expression levels in PBC688, while in G29, only 5 DEGs showed an increased expression, and the expression of the remaining 15 DEGs was significantly reduced. Interestingly, except for two novel genes (novel.7182 and novel.7237), the expression levels of the other 18 genes were significantly higher in the CMV-resistant PBC688 compared to the CMV-susceptible G29 (Figure 9). These results suggest that there were significant differences in the gene expression patterns of the photosynthesis–antenna proteins pathway between the CMV-resistant PBC688 and the CMV-susceptible G29, which may reflect distinct response mechanisms to CMV infection in these two genotypes.

## 4. Discussion

In this study, after 14 days of CMV inoculation, the CMV-susceptible G29 exhibited severe systemic leaf symptoms, with the newest leaves being most severely affected, showing wilting and necrosis. In contrast, the CMV-resistant PBC688 did not show any apparent symptoms. An absolute quantification analysis revealed a significant increase in CMV copies in G29 after CMV inoculation, with the highest CMV copies observed in the newest leaves. In PBC688, the CMV copies were much lower compared to G29, with the CMV copies in the inoculated leaves being higher than in other upwards leaves. These results indicate that the CMV-resistant PBC688 can suppress CMV replication and partially inhibit CMV long-distance movement. Previous studies using DAS–ELISA detection found that CMV proliferation rates in the CMV-resistant materials ‘Milord’ and ‘Vania’ showed no significant difference compared to the susceptible material ‘Yolo wonder’, but they did inhibit CMV movement [32]. Conversely, CMV proliferation rates in the CMV-resistant ‘Perennial’ were significantly lower than in the CMV-susceptible ‘Yolo wonder’, and CMV was detectable in non-inoculated leaves, indicating that ‘Perennial’ exhibited resistance by inhibiting CMV proliferation but not virus movement [14]. These findings are consistent with the results of this study, suggesting that the CMV-resistant material ‘PBC688’ employs a resistance mechanism similar to ‘Perennial’. 

Initially, we selected *C. annuum* CM334 for our study, hoping to use CM334 as a bridge to minimise the background effects of interspecific differences. Inoculation identification revealed that CM334 exhibited moderate resistance to CMV. However, our analyses revealed more complex background differences among CM334, G29, and PBC688. Integrating the CM334 data complicated our search for specific resistance genes, which prompted us to shift our focus to directly analysing PBC688 and G29. But for the discussion, we quoted some of the data from CM334 as references. 

In this study the number of DEGs in G29_14dci vs G29_CK was more than twice that in PBC688_14dci vs PBC688_CK, suggesting a more pronounced impact of CMV infestation on the immune system of the CMV-susceptible G29 (Figure 3). However, the number of DEGs in CM334_14dci vs CM334_CK was the least with only 1495, of which 650 are upregulated and 845 are downregulated (data unpublished). This result suggests that the resistance of PBC688 to CMV is not only active defence but may also affect the replication process of the CMV virus through changes in specific host proteins. Previous studies have found that cultivated hosts can significantly affect the recombination of CMV [33]. WUS responds to the infection of CMV and represses virus accumulation in the central zone and peripheral zone. WUS directly represses the transcription of several S-adenosyl-L-methionine-dependent methyltransferase genes, resulting in disturbed rRNA processing and ribosome stability, which affects viral protein synthesis [34].

Multiple data analyses revealed that the CMV-susceptible G29 and the CMV-resistant PBC688 exerted resistance to CMV by regulating different genes (Figure 3). During CMV infection, G29 primarily relies on genes involved in plant–pathogen interactions and the MAPK signaling pathway to exert resistance to CMV, while PBC688 relies on genes associated with ribosomes and the photosynthesis–antenna proteins pathway to confer resistance to CMV (Figure 4 and Figure 5). During CMV infestation, the DEGs of CM334 were mainly enriched in the pathways of the photosynthesis and biosynthesis of various plant secondary metabolites (data unpublished). The preceding investigation demonstrated that a transcriptome analysis of the pepper variety ‘Zunla-1’ post-CMV inoculation unveiled a total of 2143 differentially expressed genes (DEGs) across five distinct stages. Gene ontology (GO) and a KEGG analysis revealed that these DEGs participated in stress response, defense response, and plant–pathogen interaction pathways [7]. The research conducted by Lu et al. (2012) demonstrated that gene expression alterations in tobacco plants (*Nicotiana tabacum* cv. Xanthi nc) infected with the M strain of CMV were associated with disease progression [20]. An RNA sequencing analysis revealed significant differences in gene expressions between CMV-resistant and CMV-susceptible tobacco varieties following CMV infection, potentially leading to differential resistance levels to CMV. Specifically, the study indicated that systemic symptom development involved changes in photosynthesis, pigment metabolism, and plant–pathogen interaction pathways [21]. The above results were more similar to the resistance pathways in the CMV-susceptible G29 in this study, while the resistance pathways in the CMV-resistant material PBC688 were completely different.

Viral infection disrupts the equilibrium of plant hormone signal transduction, activating hormone signaling pathways such as salicylic acid (SA), jasmonic acid (JA), and ethylene pathways, which are frequently triggered by plant immune receptors [35,36]. Upon CMV inoculation, Zunla-1 exhibited approximately 22 differentially expressed genes (DEGs) involved in various signal transduction pathways, including ethylene, auxin (IAA), JA, SA, and abscisic acid (ABA) pathways [7]. Similarly, in tobacco, three DEGs associated with the ethylene pathway were downregulated in the CMV-resistant Taiyan 8, while one of them (ETR1, Cluster-14949.230569) was upregulated in the CMV-susceptible NC82 [21]. ABA has been implicated in modulating plant defenses against various viruses, including the *Bamboo Mosaic Virus* (BaMV), the *Tobacco Necrosis Virus* (TNV), and the *Chinese Wheat Mosaic Virus* (CWMV) [37,38,39,40]. Moreover, abscisic acid negatively modulates plant defenses against the *Rice Black-Streaked Dwarf Virus* infection by suppressing the jasmonate pathway and regulating ROS levels in rice [41]. In our study, approximately 30 DEGs related to hormone signal transduction were identified, with 21 DEGs involved in the ethylene pathway and 9 DEGs associated with the ABA pathway (Figure 7). These results suggest the involvement of ethylene and ABA signaling pathways in pepper defenses against CMV.

The types of ribosomes are distinguished by their sedimentation coefficients and are divided into two types: 70S and 80S. The 70S ribosome consists of a small subunit (30S) and a large subunit (50S), primarily found in prokaryotic cells, as well as in the mitochondria and chloroplasts of eukaryotic cells. On the other hand, the 80S ribosome is composed of a small subunit (40S) and a large subunit (60S), present in the cytoplasm of eukaryotic cells [42,43]. In this study, a comparison between the CMV-resistant PBC688 and the CMV-susceptible G29 revealed differential expressions of ribosomal protein genes. Specifically, the 30S and 50S ribosomal protein genes showed lower expressions in G29, while the 40S and 60S ribosomal protein genes exhibited notably lower expression levels in PBC688 (Figure 8). This suggests that the resistance of pepper to CMV may be achieved through the reduction in ribosomal protein synthesis. In Zunla-1, many genes involved in the ribosome were enriched after 24h of CMV infection [7]. Previous research indicated that ribosomal proteins played crucial roles in plant disease resistance. For example, the plant ribosomal proteins RPL12 and RPL19 function in non-host disease resistance against bacterial pathogens [44]. Additionally, the cloned ribosomal protein GaRPL18 in disease-resistant cotton materials was associated with the salicylic acid signaling pathway and exhibits resistance to *Verticillium dahliae* [45]. The interaction between the endogenous 30S ribosomal subunit protein S11 and the CMV LS2b protein affected viral replication, infection, and gene silencing suppressor activity [46]. The knockout of the 60S ribosomal subunit protein L18 gene in rice significantly reduced the expression levels of *Rice Stripe Virus* (RSV) RNA and proteins, indicating that the translation and replication of RSV were dependent on RPL18 [47]. Silencing of the chloroplast ribosomal protein large subunit 1 (NbRPL1) inhibited the infection of the tobacco vein banding mosaic virus (TVBMV), while transient expressions of NbRPL1 have little effect on the translation of TVBMV proteins [48]. Furthermore, PR proteins in plants can enhance innate resistance by inactivating ribosomes [49].

This study demonstrated that after CMV infection, several chlorophyll a–b binding protein-related genes were significantly downregulated in the CMV-susceptible G29, while in the CMV-resistant PBC688, most chlorophyll a–b binding protein-related genes were significantly upregulated (Figure 9). This result suggests that PBC688 can achieve resistance to CMV by regulating photosynthesis. In Zunla-1, a total of 14 photosynthesis-related genes were differentially expressed after CMV infection, including the photosystem I/II reaction center subunit, chlorophyllase, and the chlorophyll a/b binding protein [7]. In this study, 16 genes related to photosynthesis in CM334 were differentially expressed after CMV infestation, including the ATP synthase subunit, the photosystem I/II reaction center subunit, and Cytochrome f (data unpublished). The light-harvesting chlorophyll a/b-binding (LHCB) proteins are the apoproteins of the light-harvesting complex of photosystem II (PSII). LHCB was involved in the response of plants to some abiotic and biotic stress. LHCB1-6 can affect the response of stomata to abscisic acid inflow, thus improving the sensitivity of Arabidopsis thaliana to drought stress when they were downregulated [50,51]. LeLhcb2 overexpression enhances the tolerance of tobacco to cold stress and reduces the photooxidation of photosystem II [52]. More recently, some studies have indicated that the levels of Lhcb4, Lhcb5, and Lhcb6 are influenced by droughts, high light and high tempreture, and oxidative environmental stress [53,54]. In a previous study, the Malus domestica Lhc gene MdLhc was reported to show different expression patterns under drought stress, and transgenic Arabidopsis and apple calli expressing MdLhcb4.3 confer resistance to osmotic and drought stress [55]. The tomato chlorophyll a/b-binding protein 1C (Cab-1C) interacts with the CMV 2b protein. By 56 days post-infection (dpi), the accumulation of the CMV coat protein (CP) in the apical leaves of transgenic tobacco plants overexpressing the Cab-1C gene was significantly lower compared to that in wild-type tobacco [56]. These results demonstrate that LHCB proteins play a significant role in regulating plant resistance to various stressors.

## 5. Conclusions

This study demonstrated distinct responses of pepper genotypes to CMV infection. The CMV-susceptible G29 exhibited severe symptoms and higher virus proliferation, while the CMV-resistant PBC688 showed minimal symptoms and a lower virus content. A transcriptome analysis suggested differing genetic pathways are involved in resistance, with potential roles for ribosomal regulation and photosynthesis. These findings support the hypothesis that CMV resistance in peppers involved complex molecular mechanisms, including the modulation of host–pathogen interactions and cellular processes. Further research is needed to elucidate specific genes and pathways contributing to resistance, offering promising avenues for breeding strategies to enhance crop resilience against CMV and other viral pathogens.

## Data Availability

The raw paired-end reads were deposited into the Sequence Read Archive of the NCBI with the BioProject accession number PRJNA1110809.

## Supplementary Materials

The following supporting information can be downloaded at: https://www.mdpi.com/article/10.3390/genes15060731/s1, Figure S1: Standard Curve for absolute quantification of CMV; Table S1: The copy number of the standard template; Table S2: Absolute quantitative analysis of CMV copies of in the apical leaves; Table S3: Absolute quantitative analysis of CMV copies of in the five consecutive leaves; Table S4: Statistics from Illumina sequencing; Table S5: Differentially Expressed Genes (DEGs) between G29_14dci vs G29_CK; Table S6: Differentially Expressed Genes (DEGs) between PBC688_14dci vs PBC688_CK; Table S7: Differentially Expressed Genes (DEGs) between PBC688_CK vs G29_CK; Table S8: Differentially Expressed Genes (DEGs) between PBC688_14dci vs G29_14dci; Table S9: Gene ontology (GO) terms associated with differentially expressed genes between PBC688_14dci and G29_14dci; Table S10: The Encyclopedia of Genes and Genomes (KEGG) pathways of differentially expressed genes (DEGs) between G29_14dci and G29 Ck; Table S11: The Encyclopedia of Genes and Genomes (KEGG) pathways of differentially expressed genes (DEGs) between PBC688_14dci and PBC688_CK; Table S12: The Encyclopedia of Genes and Genomes (KEGG) pathways of differentially expressed genes (DEGs) between PBC688_CK and G29_CK; Table S13: The Encyclopedia of Genes and Genomes (KEGG) pathways of differentially expressed genes (DEGs) between PBC688_14dci and G29_14dci.

## References

[1] Palukaitis P., Roossinck M.J. (1992). *Cucumber mosaic* *virus*. Adv. Virus Res..

[2] Jacquemond M. (2012). *Cucumber mosaic* *virus*. Adv. Virus Res..

[3] Chaim A.B., Grube R., Lapidot M., Jahn M., Paran I. (2001). Identification of quantitative trait loci associated with resistance to *cucumber mosaic virus* in *Capsicum annuum*. Theor. Appl. Genet.

[4] Kang W.H., Hoang N.H., Yang H.-B., Kwon J.-K., Jo S.H., Seo J.K., Kim K.H., Choi D., Kang B.C. (2010). Molecular mapping and characterization of a single dominant gene controlling CMV resistance in peppers (*Capsicum annuum* L.). Theor. Appl. Genet.

[5] Yao M., Li N., Wang F., Ye Z. (2013). Genetic analysis and identification of QTLs for resistance to cucumber mosaic virus in chili pepper (*Capsicum annuum* L.). Euphytica.

[6] Choi S., Lee J.H., Kang W.H., Kim J., Kang B.C. (2018). Identification of *Cucumber mosaic resistance 2* (*cmr2*) that confers resistance to a new *Cucumber mosaic virus* isolate P1 (CMV-P1) in Pepper (*Capsicum* spp.). Front. Plant Sci..

[7] Zhu C.H., Li X.F., Zheng J.Y. (2018). Transcriptome profiling using Illumina- and SMRT-based RNA-seq of hot pepper for in-depth understanding of genes involved in CMV infection. Gene.

[8] Caranta C., Pflieger S., Lefebvre V., Daubeze A., Thabuis A., Palloix A. (2002). QTLs involved in the restriction of *cucumber mosaic virus* (CMV) long-distance movement in pepper. Theor. Appl. Genet..

[9] Suzuki K., Kuroda T., Miura Y., Murai J. (2003). Screening and field trials of virus resistant sources in *Capsicum* spp.. Plant Dis..

[10] Grube R.C., Zhang Y., Murphy J.F., Loaiza-Figueroa F., Lackney V.K., Provvidenti R., Jahn M.K. (2000). New source of resistance to *Cucumber mosaic virus* in *Capsicum frutescens*. Plant Dis..

[11] Guo G.J., Wang S.B., Liu J.B., Pan B.G., Diao W.P., Ge W., Gao C.Z., Snyder J.C. (2017). Rapid identification of QTLs underlying resistance to *Cucumber mosaic virus* in pepper (*Capsicum frutescens*). Theor. Appl. Genet..

[12] Palukaitis P. (2016). Satellite RNAs and satellite viruses. Mol. Plant-Microbe Interact..

[13] Caranta C., Palloix A., Lefebvre V., Daubeze A. (1997). QTLs for a component of partial resistance to *cucumber mosaic virus* in pepper: Restriction of virus installation in host-cells. Theor. Appl. Genet..

[14] Nono-Womdim R., Gebre-Selassie K., Palloix A., Pochard E., Marchoux G. (1993). Study of multiplication of cucumber mosaic virus in susceptible and resistant *Capsicum annuum* lines. J. Ann. Appl. Biol..

[15] Dufour O., Palloix A., Selassie K.G., Pochard E., Marchoux G. (1989). The distribution of *cucumber mosaic virus* in resistant and susceptible plants of pepper. Can. J. Bot..

[16] Takahashi H., Suzuki M., Natsuaki K., Shigyo T., Hino K., Teraoka T., Hosokawa D., Ehara Y. (2001). Mapping the virus and host genes involved in the resistance response in *cucumber mosaic virus*-infected *Arabidopsis thaliana*. Plant Cell Physiol..

[17] Min H.E., Han J.H., Yoon J.B., Lee J. (2016). QTL mapping of resistance to the *Cucumber mosaic virus* P1 strain in pepper using a genotyping-by-sequencing analysis. Hortic. Environ. Biote..

[18] Kang W.-H., Seo J.-K., Chung B.N., Kim K.-H., Kang B.-C. (2012). Helicase domain encoded by *cucumber mosaic virus* RNA1 determines systemic infection of *Cmr1* in pepper. PLoS ONE.

[19] Rubiales D., Fondevilla S., Chen W., Gentzbittel L., Higgins T.J.V., Castillejo M.A., Singh K.B., Rispail N. (2015). Achievements and challenges in legume breeding for pest and disease resistance. Crit. Rev. Plant Sci..

[20] Lu J., Du Z.X., Kong J., Chen L.N., Qiu Y.H., Li G.F., Meng X.H., Zhu S.F. (2012). Transcriptome analysis of *Nicotiana tabacum* infected by *cucumber mosaic virus* during systemic symptom development. PLoS ONE.

[21] Liu D., Gong M., Zhao Q., Jiang C.H., Cheng L.R., Ren M., Wang Y.Y. (2019). Comparative transcriptome analysis reveals differential gene expression in resistant and susceptible tobacco cultivars in response to infection by *cucumber mosaic virus*. Crop. J..

[22] Marathe R., Guan Z., Anandalakshmi R., Zhao H., Dinesh-Kumar S. (2004). Study of *Arabidopsis thalianaresistome* in response to *cucumber mosaic virus* infection using whole genome microarray. Plant Mol. Biol..

[23] Choi H., Jo Y., Lian S., Jo K.M., Chu H., Yoon J.Y., Choi S.K., Kim K.H., Cho W.K. (2015). Comparative analysis of chrysanthemum transcriptome in response to three RNA viruses: *Cucumber mosaic virus*, *Tomato spotted wilt virus* and *Potato virus X*. Plant Mol. Biol..

[24] Pertea M., Kim D., Pertea G.M., Leek J.T., Salzberg S.L. (2016). Transcript-level expression analysis of RNA-seq experiments with HISAT, StringTie and Ballgown. Nat. Protoc..

[25] Pertea M., Pertea G.M., Antonescu C.M., Chang T.C., Mendell J.T., Salzberg S.L. (2015). StringTie enables improved reconstruction of a transcriptome from RNA-seq reads. Nat. Biotechnol..

[26] Liao Y., Smyth G.K., Shi W. (2014). featureCounts: An efficient general purpose program for assigning sequence reads to genomic features. Bioinformatics.

[27] Love M.I., Huber W., Anders S. (2014). Moderated estimation of fold change and dispersion for RNA-seq data with DESeq2. Genome Biol..

[28] Robinson M.D., McCarthy D.J., Smyth G.K. (2010). edgeR: A Bioconductor package for differential expression analysis of digital gene expression data. Bioinformatics.

[29] Consortium G.O. (2015). Gene Ontology Consortium: Going forward. Nucleic Acids Res..

[30] Kanehisa M., Goto S. (2000). KEGG: Kyoto encyclopedia of genes and genomes. Nucleic Acids Res..

[31] Yu G., Wang L.G., Han Y., He Q.Y. (2012). clusterProfiler: An R package for comparing biological themes among gene clusters. Omics.

[32] Nono-Womdim R., Marchoux G., Pochard E., Palloix A., Gebre-Selassie K. (1991). Resistance of pepper lines to the movement of *cucumber mosaic virus*. J. Phytopathol..

[33] Ouedraogo R.S., Pita J.S., Somda I.P., Traore O., Roossinck M.J. (2019). Impact of Cultivated Hosts on the Recombination of *Cucumber Mosaic Virus*. J. Virol..

[34] Wu H., Qu X., Dong Z., Luo L., Shao C., Forner J., Lohmann J.U., Su M., Xu M., Liu X. (2020). *WUSCHEL* triggers innate antiviral immunity in plant stem cells. Science.

[35] Pieterse C.M., Van der Does D., Zamioudis C., Leon-Reyes A., Van Wees S.C. (2012). Hormonal modulation of plant immunity. Annu. Rev. Cell Dev. Biol..

[36] Aerts N., Mendes P.M., Van Wees S.C.M. (2021). Multiple levels of crosstalk in hormone networks regulating plant defense. Plant J..

[37] Iriti M., Faoro F. (2008). Abscisic acid is involved in chitosan-induced resistance to *tobacco necrosis virus* (TNV). Plant Physiol. Biochem..

[38] Alazem M., Lin K.Y., Lin N.S. (2014). The abscisic acid pathway has multifaceted effects on the accumulation of *Bamboo mosaic virus*. Mol. Plant Microbe Interact..

[39] He L., Jin P., Chen X., Zhang T.Y., Zhong K.L., Liu P., Chen J.P., Yang J. (2021). Comparative proteomic analysis of *Nicotiana benthamiana* plants under *Chinese wheat mosaic virus* infection. BMC Plant Biol..

[40] Xie K., Li L., Zhang H., Wang R., Tan X., He Y., Hong G., Li J., Ming F., Yao X. (2018). Abscisic acid negatively modulates plant defense against *rice black-streaked dwarf virus* infection by suppressing the jasmonate pathway and regulating reactive oxygen species levels in rice. Plant Cell Environ..

[41] Wool I.G., Chan Y.L., Glück A. (1995). Structure and evolution of mammalian ribosomal proteins. Biochem. Cell Biol..

[42] Wang B., Song N., Tang C., Ma J., Wang N., Sun Y., Kang Z. (2019). PsRPs26, a 40S Ribosomal protein subunit, regulates the growth and pathogenicity of *Puccinia striiformis* f. sp. Tritici. Front. Microbiol..

[43] Nagaraj S.S., Senthil-Kumar M., Ramu V.S., Wang K., Mysore K.S. (2016). Plant ribosomal proteins, RPL12 and RPL19, play a role in non-host disease resistance against bacterial pathogens. Front. Plant Sci..

[44] Gong Q., Yang Z., Wang X., Butt H.I., Chen E., He S., Zhang C., Zhang X., Li F. (2017). Salicylic acid-related cotton (*Gossypium arboreum*) ribosomal protein GaRPL18 contributes to resistance to *Verticillium dahliae*. BMC Plant Biol..

[45] Wang R., Du Z., Bai Z., Liang Z. (2017). The interaction between endogenous 30S ribosomal subunit protein S11 and *Cucumber mosaic virus* LS2b protein affects viral replication, infection and gene silencing suppressor activity. PLoS ONE.

[46] Li S., Li X., Zhou Y. (2018). Ribosomal protein L18 is an essential factor that promote rice stripe virus accumulation in small brown planthopper. Virus Res..

[47] Cheng D.J., Xu X.J., Yan Z.Y., Tettey C.K., Fang L., Yang G.L., Geng C., Tian Y.P., Li X.D. (2021). The chloroplast ribosomal protein large subunit 1 interacts with viral polymerase and promotes virus infection. Plant Physiol..

[48] Islam M.M., El-Sappah A.H., Ali H.M., Zandi P., Huang Q., Soaud S.A., Alazizi E.M.Y., Wafa H.A., Hossain M.A., Liang Y. (2023). Pathogenesis-related proteins (PRs) countering environmental stress in plants: A review. S. Afr. J. Bot.

[49] Xu Y.H., Liu R., Yan L., Liu Z.Q., Jiang S.C., Shen Y.Y., Wang X.F., Zhang D.P. (2012). Light-harvesting chlorophyll a/b-binding proteins are required for stomatal response to abscisic acid in *Arabidopsis*. J. Exp. Bot..

[50] Liu R., Xu Y.H., Jiang S.C., Lu K., Lu Y.F., Feng X.J., Wu Z., Liang S., Yu Y.T., Wang X.F. (2013). Light-harvesting chlorophyll a/b-binding proteins, positively involved in abscisic acid signalling, require a transcription repressor, WRKY40, to balance their function. J. Exp. Bot..

[51] Deng Y.-S., Kong F.Y., Zhou B., Zhang S., Yue M.M., Meng Q.W. (2014). Heterology expression of the tomato LeLhcb2 gene confers elevated tolerance to chilling stress in transgenic tobacco. Plant Physiol. Biochem..

[52] Chen Y.E., Liu W.J., Su Y.Q., Cui J.M., Zhang Z.W., Yuan M., Zhang H.Y., Yuan S. (2016). Different response of photosystem II to short and long-term drought stress in Arabidopsis thaliana. Physiol. Plant.

[53] Chen Y.E., Zhang C.M., Su Y.Q., Ma J., Zhang Z.W., Yuan M., Zhang H.Y., Yuan S. (2017). Responses of photosystem II and antioxidative systems to high light and high temperature co-stress in wheat. Environ. Exp. Bot..

[54] Chen Y.E., Ma J., Wu N., Su Y.Q., Zhang Z.W., Yuan M., Zhang H.Y., Zeng X.Y., Yuan S. (2018). The roles of Arabidopsis proteins of Lhcb4, Lhcb5 and Lhcb6 in oxidative stress under natural light conditions. Plant Physiol. Biochem..

[55] Zhao S., Gao H., Luo J., Wang H., Dong Q., Wang Y., Yang K., Mao K., Ma F. (2020). Genome-wide analysis of the light-harvesting chlorophyll a/b-binding gene family in apple (Malus domestica) and functional characterization of MdLhcb4.3, which confers tolerance to drought and osmotic stress. Plant Physiol. Biochem..

[56] Liu Z., Yu C., Xiang B., Niu J., Zheng Y. (2022). Processing tomato chlorophyll a/b-binding protein 1C interacts with CMV 2b protein. Physiol. Mol. Plant P.

