# Supplementary figures and images for: Transcriptional Comparison Reveals Differential Resistance Mechanisms between CMV-Resistant PBC688 and CMV-Susceptible G29
Source: Genes (Basel). 2024 Jun 2;15(6):731. doi: 10.3390/genes15060731 (PMC11202605; doi:10.3390/genes15060731)

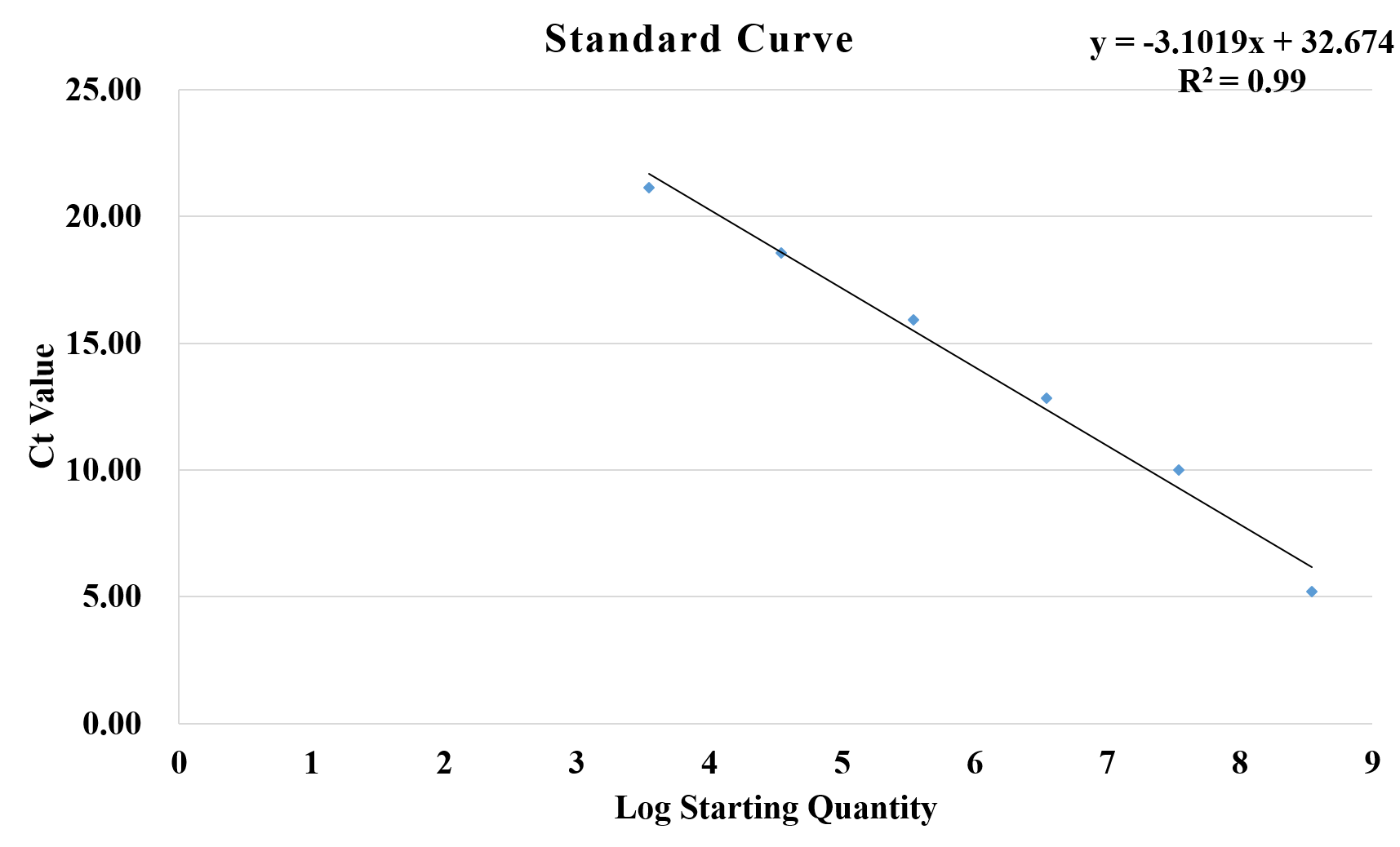

Supplement: Supplementary file 1 [file genes-15-00731-s001.zip › Supplementary Figure 1.tif]
